# Effect of Pioglitazone on the Course of New-Onset Type 1 Diabetes Mellitus

**DOI:** 10.4274/Jcrpe.981

**Published:** 2013-12-12

**Authors:** Kimberly Sue Tafuri, Mushtaq Ahmed Godil, Andrew Harry Lane, Thomas Allen Wilson

**Affiliations:** 1 Division of Pediatric Endocrinology, Stony Brook Children’s Hospital, Stony Brook, United States; 2 Division of Pediatric Endocrinology, Geisinger Health System, Danville, United States

**Keywords:** type 1 diabetes, children, thiazolidinedione, β cell function

## Abstract

**Objective:** Type 1 diabetes mellitus (T1DM) is caused by insulin deficiency resulting from progressive destruction of β cells. The histological hallmark of the diabetic islet is mononuclear cell infiltration. Thiazolidinediones (TZDs) activate PPARg and enhance the actions of insulin. Studies in non-obese diabetic and streptocotozin-treated mouse models demonstrated that pretreatment with TZDs prevented the development of T1DM. The purpose of this study was to examine whether pioglitazone, given with insulin, preserved β cell function in patients with new-onset T1DM.

**Methods:** This was a randomized, double-blind, placebo-controlled 24-week study. Subjects received pioglitazone or placebo. Blood sugar, glycated hemoglobin (HbA1c), C-peptide, and liver enzymes were measured at baseline. Boost© stimulated C-peptide responses were measured at baseline and at 24 weeks. Blood sugar, insulin dose, height, weight, and liver enzymes were monitored at each visit. HbA1c was performed every 12 weeks.

**Results:** Of the 15 patients, 8 received pioglitazone, and 7 - placebo. There was no clinical improvement in HbA1c between or within groups at the completion of the study. Mean peak C-peptide values were similar between groups at baseline. Mean peak C-peptide level was slightly higher at 24 weeks in the pioglitazone group compared to the placebo (1.8 vs. 1.5

ng/mL) which was considered as clinically insignificant. The interaction of HbA1c and insulin dose (HbA1c* insulin/kg/day), which combines degree of diabetic control and dose of insulin required to achieve this control, showed transient improvement in the pioglitazone group at 12 weeks but was not sustained at 24 weeks.

**Conclusion:** In this pilot study, pioglitazone did not preserve β cell function when compared to placebo.

**Conflict of interest:**None declared.

## INTRODUCTION

Type 1 diabetes is mellitus (T1DM) a common chronic disease in children and young adults. It is most common before the age of 20, although it can occur at any age (1). It is considered to be a prototypic autoimmune disease, caused by progressive and selective destruction of the insulin-producing β cells of islets of Langerhans resulting in absolute insulin deficiency (2).

The histological hallmark of the diabetic islet is mononuclear cell infiltration or insulitis. Studies on the post-mortem diabetic pancreas reveal that the islet cell infiltrate is comprised primarily of T-cells although a significant number of β cells and macrophages are also present (3,4). Studies have also suggested that cytotoxic cytokines such as tumor necrosis factor alpha (TNF-a) and interleukin (IL)-1β also contribute to the destruction of the pancreatic β cell (5).

Thiazolidinediones (TZDs) are a class of drugs used in the treatment of T2DM. These agents activate PPARg and enhance the actions of insulin thereby increasing glucose uptake into the muscle and reducing hepatic glucose output (6). TZDs have also been shown to decrease pancreatic β cell destruction (6,7,8,9) and therefore have a potential role in the treatment and prevention of T1DM.

Commonly used murine models of T1DM are non-obese diabetic (NOD) and multiple low-dose streptocotozin (MLDS)-induced diabetes mice. To date, multiple studies in the mouse model have demonstrated a decrease in the incidence of diabetes when pretreated with TZDs (5,8,9,10,11). Furthermore, in vitro studies of human islets cultured with pioglitazone prevented apoptosis of the β cell caused by IL-1β and high glucose concentrations. The protective effects of pioglitazone on the β cell were believed to be via blockade of the NFkB pathway (12).

Based on these observations, we hypothesized that the use of TZDs in newly diagnosed patients with T1DM might preserve remaining β cell function, reduce insulin requirement and prolong the honeymoon period. 

## METHODS

This was a prospective, randomized, double-blind, placebo-controlled 24 week study. Fifteen subjects with newly diagnosed T1DM were enrolled within 4 months of diagnosis. Subjects were included if they met the following criteria: age >6 years and positive immune markers for T1DM. Children with a history of underlying liver disease were excluded. Informed consent was obtained from the parents of all children and assent was obtained from children >12 years of age. The study was approved by the Stony Brook University IRB and registered in ClinicalTrials.Gov (# NCT00545857).

Subjects were randomized to receive either pioglitazone or placebo. The dose of pioglitazone was as follows: 15

mg/day for children 6-10 years old, 30 mg/day for children

10-15 years old and 45 mg/day for children older than 15 years old. Patients were followed at baseline, 2 and 4 weeks, and monthly thereafter for a total of 24 weeks. A blood sugar and hepatic panel was measured at baseline and at each subsequent visit. Boost© stimulated C-peptide and blood sugar were measured at baseline, 30, 60, 90, and 120 minutes prior to the start of the study and again at 24 weeks. Glycated hemoglobin (HbA1c) was measured at baseline and every 12 weeks. Height, weight, and insulin dose were recorded at each visit. The dose of insulin was adjusted in an attempt to maintain the blood sugar in the target range of 80-180 mg/dL.

If the liver enzymes increased more than two fold, the study drug (pioglitazone or placebo) was discontinued and the subject was removed from the study. Post-menarchal females had a qualitative serum HCG assessment at baseline, 12 weeks and 24 weeks. A positive HCG resulted in removal of the patient from the study.

Blood samples were drawn at the General Clinical Research Center (GCRC). Samples for C-peptide were spun promptly and frozen at -20˚C. All samples from an individual patient were batched and run in the same assay after the 24-week samples were obtained. C-peptide was measured by ARUP laboratories using a Quantitative Chemiluminescent Immunoassay. The lower limit of detection was 0.1 ng/mL. Determinations of HbA1c, blood sugar and a hepatic panel were performed by the University Hospital clinical laboratory.

**Statistical Analysis**

Due to the small sample size, no statistical analyses were performed in this pilot study. The data are reported as mean±standard deviation values. 

## RESULTS

Fifteen patients were enrolled - 8 received pioglitazone and 7 placebo. Six of the eight subjects in the pioglitazone group completed the study. One patient was removed because of a transient elevation in liver enzymes and one voluntarily withdrew without giving a specific reason.

**Figure 1:**
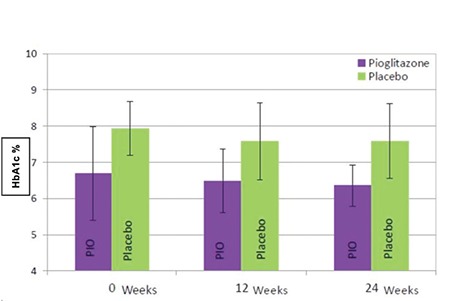
Comparison of HbA1c at baseline, 12 and 24 weeks between groups. Bars represent 1 standard deviation

Demographic and clinical data are displayed in Table 1. Age at entry and duration of diabetes were similar between the treatment and placebo groups. Baseline daily insulin dose was higher in the pioglitazone group (0.46 u/kg/day vs. 0.28

u/kg/day). Baseline HbA1c was slightly lower in the pioglitazone group [6.7% (50 mmol/mol) vs. 7.9% (63

mmol/mol)]. HbA1c did not change substantially within groups at the completion of the study [pioglitazone: 6.4% (46 mmol/mol) vs. placebo: 7.6% (60 mmol/mol)] (Figure 1).

**Figure 2:**
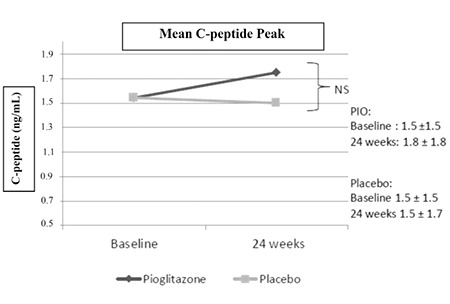
Comparison of mean peak C-peptide levels at baseline and 24 weeks. Mean±standard deviation listed for both groups

Mean peak C-peptide values were similar between groups at baseline (pioglitazone: 1.5 vs. placebo: 1.5 ng/mL). Mean peak C-peptide was slightly greater at 24 weeks in the pioglitazone group compared to placebo (1.8 vs 1.5 ng/mL) which was assessed to be clinically insignificant (Figure 2).

**Figure 3:**
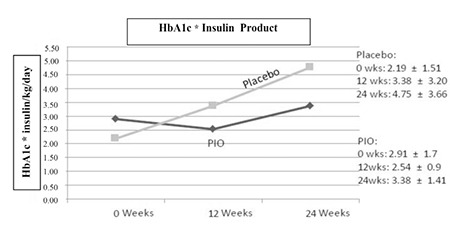
Interaction of HbA1c and insulin dose. Mean±standard deviation listed for both groups

The interaction of HbA1c and insulin dose (HbA1c* insulin/kg/day), which combines degree of diabetic control and dose of insulin required to achieve this control, showed transient improvement in the pioglitazone group at 12 weeks but was not sustained at 24 weeks, whereas the placebo group demonstrated a progressive increase throughout the 24 weeks (Figure 3).

No adverse events were encountered. None of the patients developed edema or heart failure during the study period. There was no significant difference in change in body mass index or weight between the groups over the 24 weeks. One child in the pioglitazone group developed transient elevation in liver enzymes which resolved after pioglitazone was discontinued.

## DISCUSSION

Multiple diabetic mouse models have demonstrated clinical improvement of diabetes when pretreated with TZDs. Takamura et al (8) investigated the effect of pioglitazone, a TZD, on the development of MLDS-induced autoimmune diabetes in mice. MLDS injected intraperitoneally resulted in mononuclear cell infiltration in and around the islets, followed by hyperglycemia. Oral administration of pioglitazone prevented or delayed the development of diabetes induced by MLDS. Histologically, pioglitazone blocked the infiltration of mononuclear cells into islets in MLDS mice (8). These findings suggest that pioglitazone blocked the autoimmune process in the development of MLDS diabetes. Similarly, Beales et al (10) prevented the development of diabetes in the NOD mouse with pretreatment with the TZD, troglitazone. In another study in MLDS-injected mice, treatment with troglitazone prevented hyperglycemia, suppressed insulitis and inhibited TNF-a production from intraperitoneal exudate cells (5).

Our study is unique in that it is the only study examining the effect of TZDs in children after initial diagnosis of T1DM when residual β cell function is expected to be present. Unlike the studies conducted in the NOD mouse model, our study did not demonstrate clinical improvement in β cell function. This may be related to the difference in the composition of pancreatic cellular infiltrate in the NOD mice and humans, which predominantly consists of CD 4 T-cells in NOD mice vs. CD 8 T-cells in humans (13). Although the mean duration of diabetes from diagnosis was only 3.1 months, our failure to demonstrate clinical improvement in β cell function may be due to the fact that the majority of β cell destruction occurred prior to the manifestation of clinical symptoms.

There was a transient improvement in the interaction of HbA1c and insulin dose (as reflected in the HbA1c* insulin dose product) at 12 weeks, but this was not sustained at 24 weeks in the pioglitazone group. When the 2 subjects who dropped out of the pioglitazone group after 12 weeks were excluded from the analysis, the conclusions did not change. The failure to maintain an improved HbA1c* insulin dose product may have been impacted by the lower doses of pioglitazone used in this study (in terms of dose per kg body weight) compared to what was used in animal models. A study conducted in T2DM adults demonstrated that pioglitazone, at doses of 30 or 45 mg/day, caused a dose-dependent decrease in fasting and postprandial blood glucose concentrations through improvements in hepatic/whole-body insulin sensitivity and β cell function (14). In our study, the majority of patients received either 15 or 30 mg of pioglitazone. It is possible that the failure to maintain HbA1c* insulin may have been impacted by the lower doses of pioglitazone used in this study, but more likely is due to the differences in β cell function between T1DM and T2DM.

Our negative findings are supported by Shigihara et al (15) who showed no improvement in insulitis or cytokine profile in NOD mice treated with pioglitazone when compared to metformin. Similarly, Zdravkovic et al (16) failed to show improvement in HbA1c or a decrease in insulin requirements in obese adolescents with T1DM treated with pioglitazone. Bhat et al (17) were able to demonstrate a modest improvement in HbA1c and post prandial blood glucose levels in lean type 1 adolescents treated with pioglitazone; however, there was no change in insulin requirement.

Studies conducted by Shimada et al (18) in adults with slowly progressive T1DM showed progression of disease in subjects treated with pioglitazone when compared to those receiving metformin. At two years post intervention, 3 of the 4 subjects in the pioglitazone group reached a HbA1c of 8% compared to 1 of 5 in the metformin group (18). Shimada et al (18) reported that pioglitazone accelerates disease progression, but this conclusion may have been impacted by the small sample size.

In contrast, rosiglitazone alone and rosiglitazone combined with insulin was found to preserve endogenous C-peptide secretion and percent β cell function (HOMA2-%B) in adults with latent autoimmune diabetes when compared to insulin or sulfonylureas, suggesting a potential benefit of TZDs on β cell function (19).

Increasing evidence in the mouse model supports a role for TZDS in the prevention of T1DM; however, its utility in preserving β cell function in children and adults with T1DM remains doubtful.

In conclusion, in this pilot study, pioglitazone did not appear to preserve β cell function in children with T1DM when compared to placebo. Larger, long-term prospective studies need to be conducted to identify the potential beneficial effects, if any, of pioglitazone and other TZDs on preservation of β cell function in T1DM.

## ACKNOWLEDGEMENTS

The protocol was approved by the institutional review board and the GCRC. Grant support was provided by the GCRC (grant MO1RR10710). 

Guarantor: Thomas Allen Wilson MD.

## Figures and Tables

**Table 1:**
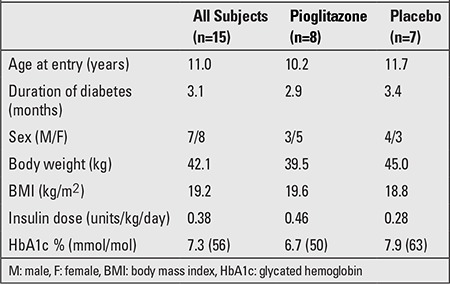
Demographics and clinical data in the patients (mean values)
